# Supplementary material for: Weather impacts expressed sentiment
Source: PLoS One. 2018 Apr 25;13(4):e0195750. doi: 10.1371/journal.pone.0195750 (PMC5918636; doi:10.1371/journal.pone.0195750)
Supplement: S1 File — Equation A in S1 File: User-level regression equation. Fig A in S1 File: Twitter user-level analyses for all message types. Fig B in S1 File: Twitter user-level analyses for posts without weather terms. Fig C in S1 File: User-level comparisons between the effect size of below freezing temperatures on positive, non-weather, expressed sentiment with the effect sizes of other locale-specific events over the course of our data on the same sentiment metric. Fig D in S1 File: Effects of the weather on city-level frequency and user-level probability of weather speech. Fig E in S1 File: Effects of the weather on expressed sentiment of posts that contain weather terms. Fig F in S1 File: Effect sizes in context for negative sentiment. Fig G in S1 File: Replication using SentiStrength classification. Fig H in S1 File: Replication using SentiStrength classification. Fig I in S1 File: Replication using Hedonometer classification. Fig J in S1 File: Replication using Hedonometer classification. Fig K in S1 File: Correlation between alternative positive sentiment state metrics. Fig L in S1 File: Correlation between alternative positivenegative sentiment state metrics. Table A in S1 File: Summary statistics of main dependent and independent variables. Table B in S1 File: Examples of LIWC sentiment encoding. Table C in S1 File: City-level weather and expressed sentiment, all posts. Table D in S1 File: User-level weather and expressed sentiment, Twitter all posts. Table E in S1 File: City-level weather and expressed sentiment, Twitter no weather terms. Table F in S1 File: User-level weather and expressed sentiment, Twitter no weather terms. (PDF) [file pone.0195750.s001.pdf]

Weather impacts expressed sentiment

Supporting Information

Patrick Baylis, Nick Obradovich, Yury Kryvasheyeu, Haohui Chen, Lorenzo Coviello, Esteban Moro, Manuel Cebrian, James H. Fowler

Contents

User-level analysis **2**

Methods . . . 2

Descriptive statistics . . . 2

All expressed sentiment . . . 2

No weather terms . . . 3

Effect sizes in context . . . 3

LIWC sentiment examples **3**

Weather terms **4**

Rate of messages containing weather terms **4**

Sentiment of messages containing weather terms **8**

Effect size context for negative sentiment **8**

Regression tables **8**

All posts . . . 8

Posts without weather terms . . . 8

Alternative measures of expressed sentiment **13**

SentiStrength . . . 13

Hedonometer . . . 13

Correlation of classifiers of expressed sentiment . . . 17

References **19**

User-level analysis

Methods

For our user-level analysis, we employ the posts of users who authored Twitter messages on greater than 25% of days in our sample, a subsample containing 511 million tweets across 365,476 users. We calculate our expressed sentiment dependent variables identically to the city-level analysis, stopping at the user-level of aggregation. For each user-day we have the percentage of that user's tweets that contain positive sentiment as well as the percentage of the user's tweets that contain negative sentiment. We restrict our user-level analysis to our Twitter data as we no longer retain access to the user-level Facebook data.

To investigate if the weather is associated with alterations of expressed sentiments within individuals over time, we employ our user-level data, along with a slightly modified equation from the main text. We model our user-level relationship as:

$$Y_{ijmt} = f(tmax_{ijmt}) + g(precip_{ijmt}) + h(\mu) + \eta_i + \gamma_t + \nu_{jm} + \epsilon_{ijmt} \qquad (A)$$

In Equation A i now indexes unique individuals and η_i replaces α_j and represents user-level indicator terms that control for individual-specific, time-invariant factors such as average mood, constant demographic covariates, and fixed weather preferences [1]. The model again includes calendar date and city-level by year-month indicator terms.

Descriptive statistics

Table A: Summary statistics of main dependent and independent variables.

	Twitter User Mean	Twitter User Std. Dev.
Pos. Rate	33.32	35.02
Neg. Rate	18.13	27.52
Pos. No Wth.	32.99	35.06
Neg. No Wth.	18.1	27.64
Max. Temperature	21.69	10.32
Precipitation	0.27	0.83
Cloud Cover	39.8	26.88
Humidity	68.39	16.7

We present the descriptive statistics associated with our main user-level variables in Table A.

All expressed sentiment

Panels (a) and (b) of Fig A display the results of estimating Equation A on 81,388,085 user-days of Twitter user-level data. The nature of the impact of temperature and precipitation on sentiment expression is quite similar to the effect size in the city-level data, though these are again attenuated in magnitude. The effect sizes of precipitation, temperature range, and cloud cover on user-level expressed sentiment on Twitter retain statistical significance but are similarly attenuated in magnitude as compared to the city-level Twitter model (see *Tables for Fig 1* for details). The association between high levels of humidity and positive expressed sentiment fails to gain significance in this model.

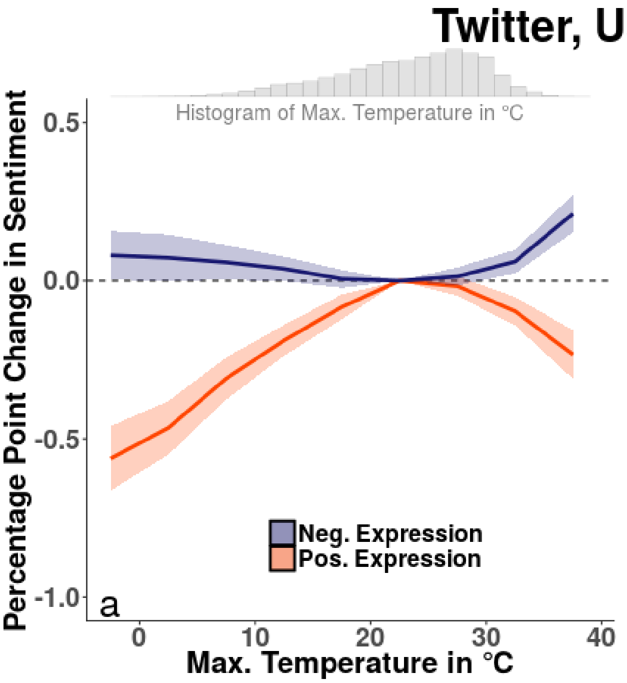

Fig A: **Twitter user-level analyses for all message types.** Panels (a) and (b) replicate the main text analyses for Twitter user-level data. Shaded error bounds represent 95% confidence intervals.

No weather terms

Excluding weather terms from the user-level data also reduces effect sizes somewhat, as can be seen for temperature and precipitation in panels (a) and (b) of Fig B. In this model, the association between cold temperatures and large amounts of precipitation and negative expressed sentiment fail to gain statistical significance (though more moderate amounts of precipitation still significantly associate with increased rates of negative sentiment). High temperature ranges in this model still associate with improved expressed sentiment while cloud cover again associates with worsened sentiment. Humidity fails to gain significance, though the signs of the associations remain the same.

Effect sizes in context

At the user level, the effect sizes associated with the weather are smaller than at the city-level but are still meaningful (see Fig C). At this level, a day of below freezing temperature is associated with 37% the effect size of the Carolina flooding on user-level expressed sentiment in Charlotte.

LIWC sentiment examples

Table B displays an example of user posts from a day, encoded with our LIWC sentiment classification tool. Posts are aggregated as described in our Methods section in the main text.

Table B: Examples of LIWC sentiment encoding.

Text	Positive	Negative
This line is soo terribly long. I hate it!	0	1
That movie was awesome! I'm excited thinking about it.	1	0

Text	Positive	Negative
I slept awful last night and am soooo tired	0	1
This smoothie takes ten minutes to make, it's worth it.	0	0
Daily Average:	0.25	0.5

Weather terms

Below we list the 318 crowd-sourced weather terms that we exclude at the tweet level in order to calculate our non-weather related sentiment metrics.

aerovane air airstream altocumulus altostratus anemometer anemometers anticyclone anticyclones arctic arid aridity atmosphere atmospheric autumn autumnal balmy baroclinic barometer barometers barometric blizzard blizzards blustering blustery blustery breeze breezes breezy brisk calm celsius chill chilled chillier chilliest chilly chinook cirrocumulus cirrostratus cirrus climate climates cloud cloudburst cloudbursts cloudier cloudiest clouds cloudy cold colder coldest condensation contrail contrails cool cooled cooling cools cumulonimbus cumulus cyclone cyclones damp damp damper damper dampest dampest degree degrees deluge dew dews dewy doppler downburst downbursts downdraft downdrafts downpour downpours dried drier dries driest drizzle drizzled drizzles drizzly drought droughts dry dryline fall farenheit flood flooded flooding floods flurries flurry fog fogbow fogbows fogged fogging foggy fogs forecast forecasted forecasting forecasts freeze freezes freezing frigid frost frostier frostiest frosts frosty froze frozen gale gales galoshes gust gusting gusts gusty haboob haboobs hail hailed hailing hails haze hazes hazy heat heated heating heats hoarfrost hot hotter hottest humid humidity hurricane hurricanes ice iced ices icing icy inclement landspout landspouts lightning lightnings macroburst macrobursts maelstrom mercury meteorologic meteorologist meteorologists meteorology microburst microbursts microclimate microclimates millibar millibars mist misted mists misty moist moisture monsoon monsoons mugginess muggy nexrad nippy NOAA nor'easter nor'easters noreaster noreasters overcast ozone parched parching pollen precipitate precipitated precipitates precipitating precipitation psychrometer radar rain rainboots rainbow rainbows raincoat raincoats rained rainfall rainier rainiest raining rains rainy sandstorm sandstorms scorcher scorching searing shower showering showers skiff sleet slicker slickers slush slushy smog smoggier smoggiest smoggy snow snowed snowier snowiest snowing snowmageddon snowpocalypse snows snowy spring sprinkle sprinkles sprinkling squall squalls squally storm stormed stormier stormiest storming storms stormy stratocumulus stratus subtropical summer summery sun sunnier sunniest sunny temperate temperature tempest thaw thawed thawing thaws thermometer thunder thundered thundering thunders thunderstorm thunderstorms tornadic tornado tornadoes tropical troposphere tsunami turbulent twister twisters typhoon typhoons umbrella umbrellas vane warm warmed warming warms warmth waterspout waterspouts weather wet wetter wettest wind windchill windchills windier windiest windspeed windy winter wintery wintry

Rate of messages containing weather terms

Below we analyze the effect of meteorological conditions on the rate of expressions that contain weather terms. As can be seen in Fig D, city-level rates and individual-level probabilities of weather speech, unsurprisingly, increase and comprise a larger percentage of overall expressions under less pleasant meteorological conditions.

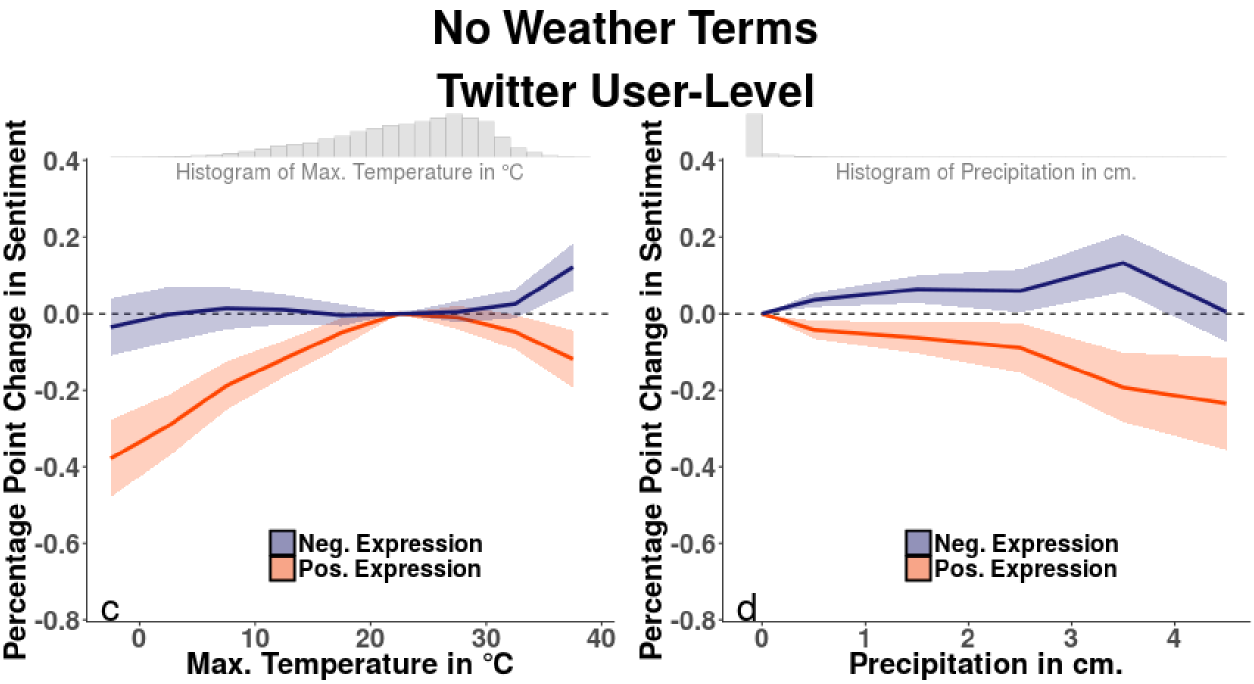

Fig B: **Twitter user-level analyses for posts without weather terms.** Panels (a) and (b) depict the results of estimating Equation S2 on the sentiment of non-weather posts at the user level. Shaded error bounds represent 95% confidence intervals.

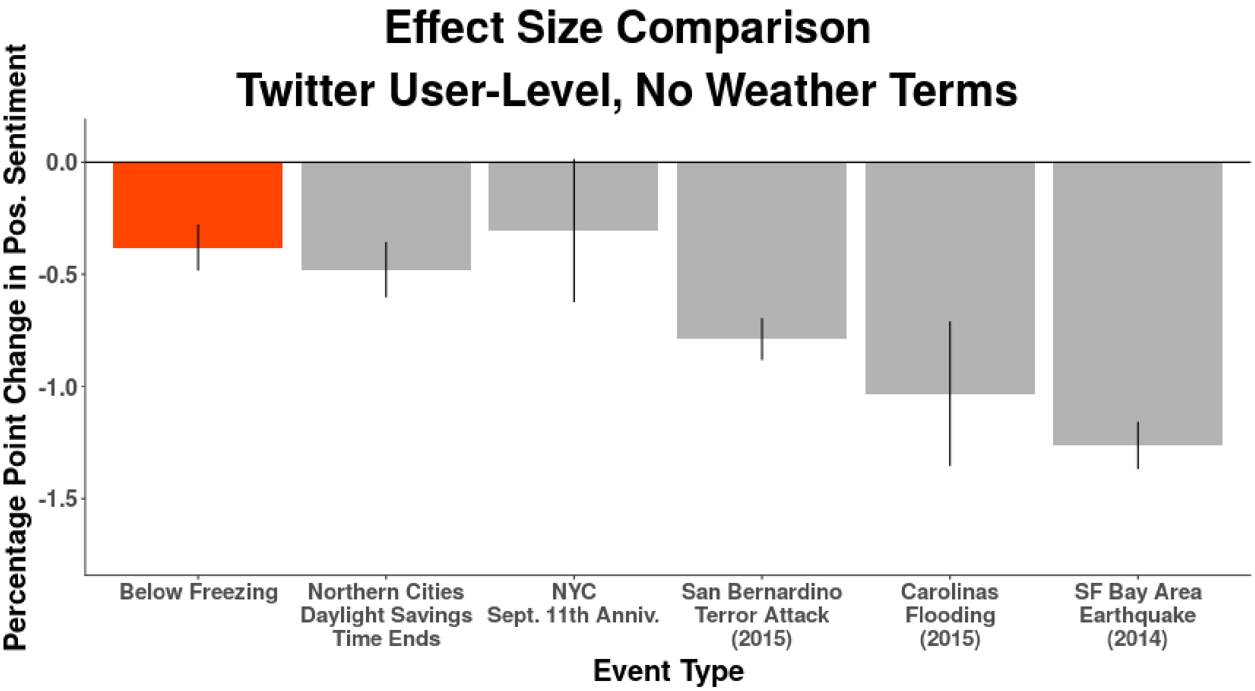

Fig C: **User-level comparisons between the effect size of below freezing temperatures on positive, non-weather, expressed sentiment with the effect sizes of other locale-specific events over the course of our data on the same sentiment metric.** The effect size of freezing temperatures compares in magnitude to other significant events.

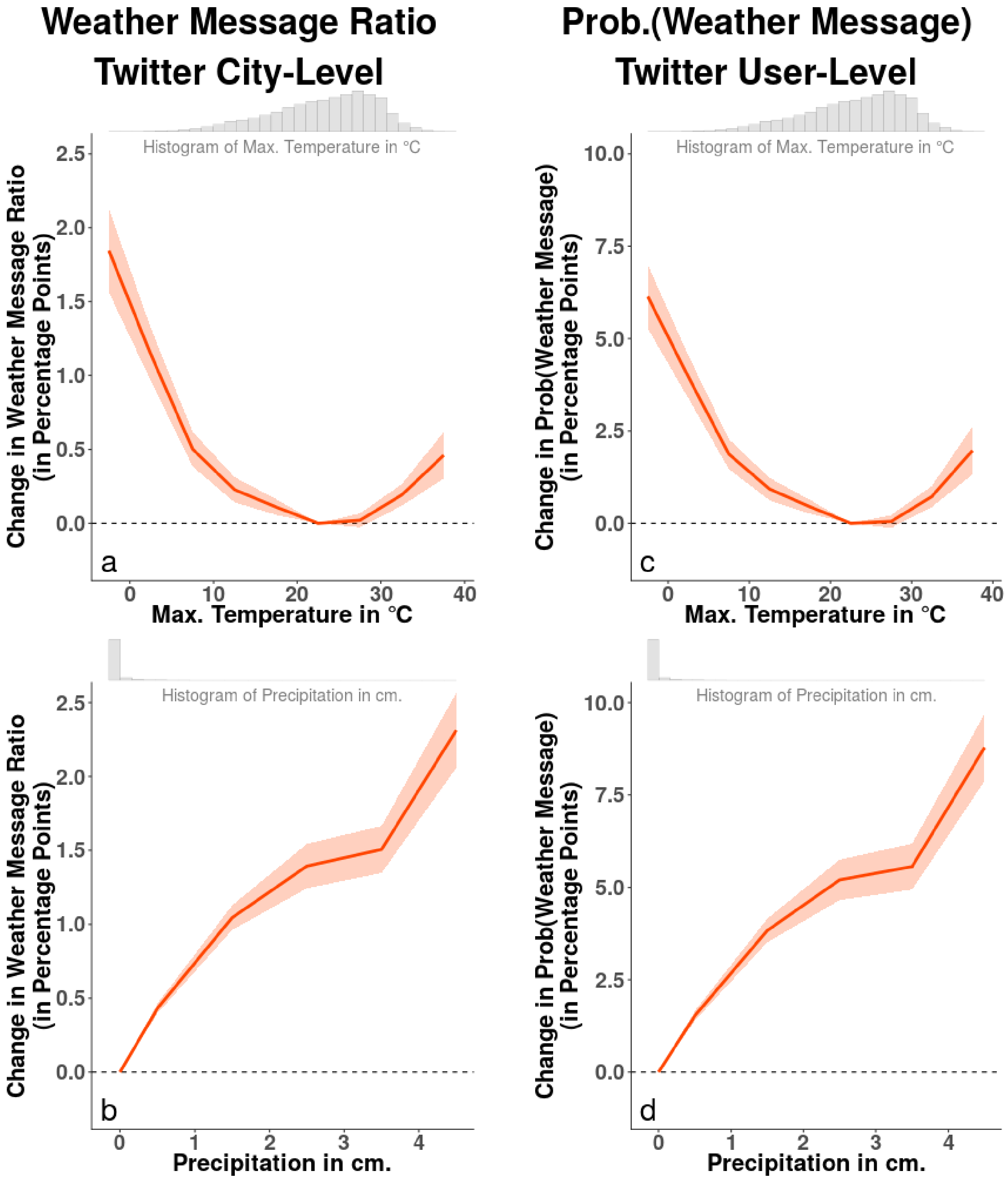

Fig D: **Effects of the weather on city-level frequency and user-level probability of weather speech.**

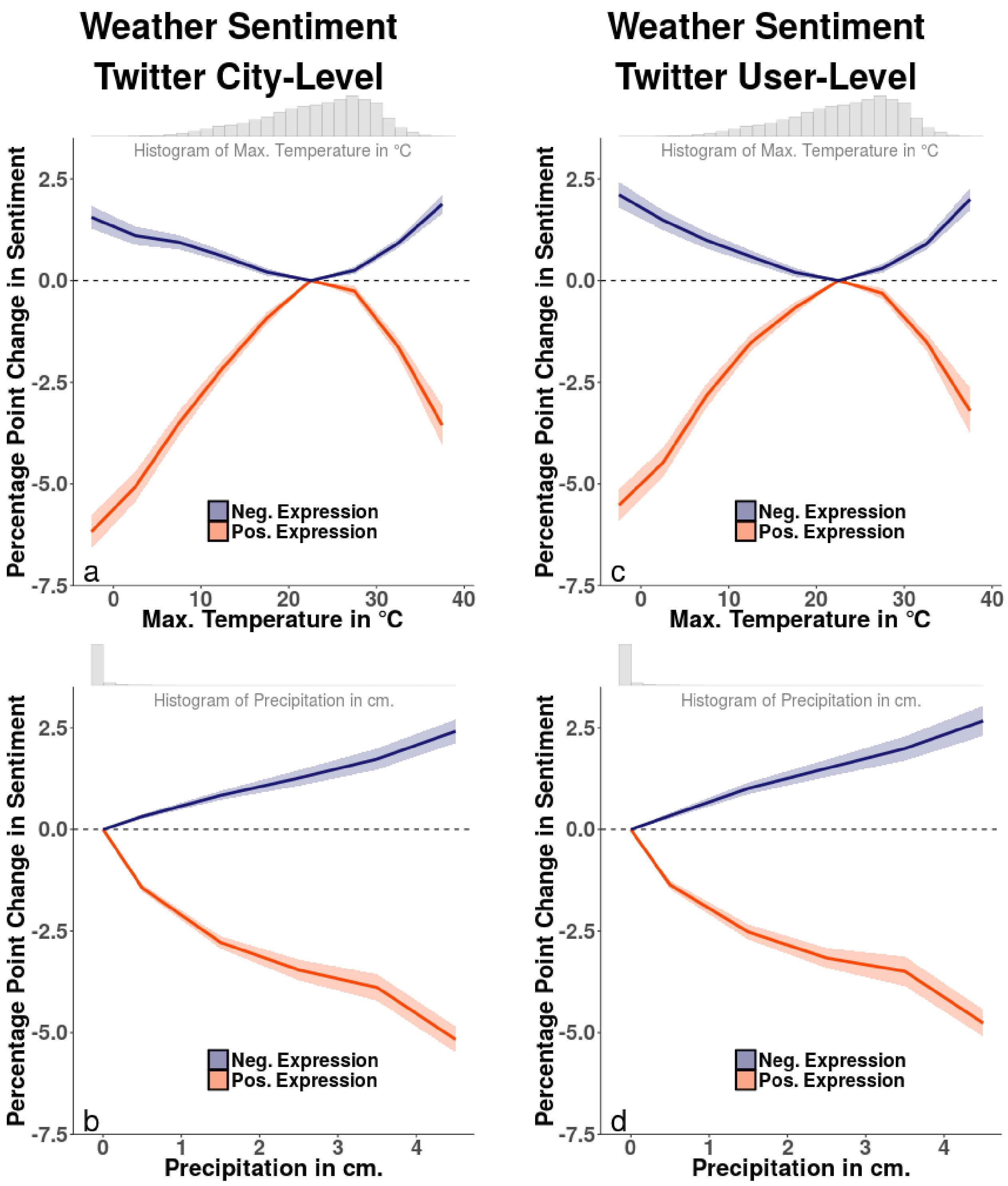

Fig E: **Effects of the weather on expressed sentiment of posts that contain weather terms.**

Sentiment of messages containing weather terms

We also analyze the effect of meteorological conditions on the sentiment expressions that contain weather terms. As can be seen in Fig E, again unsurprisingly, both city-level and individual-level expressed sentiment of weather messages markedly worsens under less pleasant meteorological conditions.

Effect size context for negative sentiment

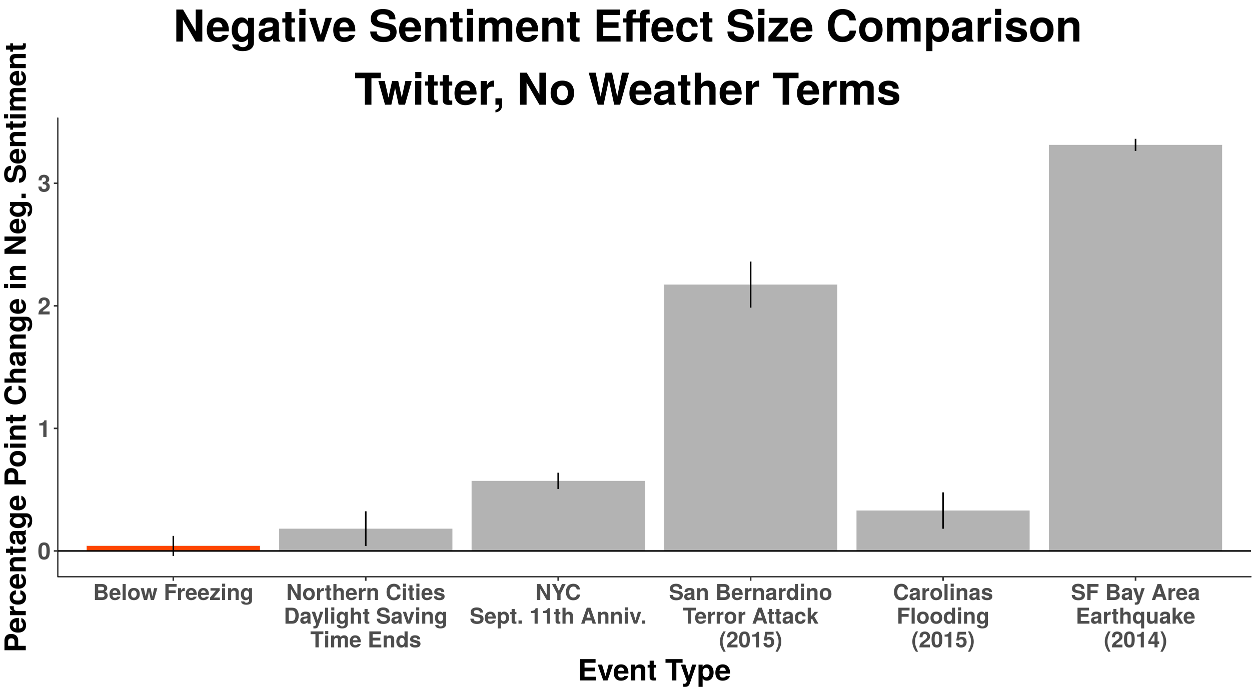

Fig F: **Effect sizes in context for negative sentiment.** Comparisons between the effect size of below freezing temperatures on negative, non-weather, expressed sentiment with the effect sizes of other locale-specific events over the course of our data on the same sentiment metric at the Twitter city-level. Error bars represent 95% confidence intervals calculated using heteroskedasticity-robust standard errors clustered on both city-year-month and day.

Fig F presents the replication of Fig 3 from the main text employing negative rather than positive sentiment.

Regression tables

All posts

Table C corresponds to the city-level results associated with Equation 1 and Fig 1 from the main text. Table D corresponds to the user-level results presented above.

Posts without weather terms

Table E corresponds to the city-level results associated with Equation 1 and Fig 2 from the main text. Table F corresponds to the user-level, no weather results presented above.

Table C: City-level weather and expressed sentiment, all posts

	Dependent variable:			
	Positive	Negative	Positive	Negative
	Facebook		Twitter	
	(1)	(2)	(3)	(4)
cuttmax(-Inf,0]	−1.647***	0.362***	−0.737***	0.109**
	(0.097)	(0.075)	(0.054)	(0.045)
cuttmax(0,5]	−1.413***	0.251***	−0.580***	0.138***
	(0.081)	(0.055)	(0.042)	(0.036)
cuttmax(5,10]	−1.034***	0.246***	−0.436***	0.147***
	(0.062)	(0.040)	(0.035)	(0.028)
cuttmax(10,15]	−0.693***	0.184***	−0.280***	0.109***
	(0.043)	(0.028)	(0.025)	(0.020)
cuttmax(15,20]	−0.313***	0.110***	−0.115***	0.048***
	(0.025)	(0.020)	(0.019)	(0.014)
cuttmax(25,30]	0.073***	−0.046**	−0.010	0.016
	(0.025)	(0.020)	(0.018)	(0.015)
cuttmax(30,35]	−0.216***	0.055*	−0.083***	0.066***
	(0.037)	(0.029)	(0.024)	(0.019)
cuttmax(35, Inf]	−0.720***	0.232***	−0.278***	0.195***
	(0.063)	(0.039)	(0.037)	(0.029)
cutprcp(0,1]	−0.212***	0.117***	−0.099***	0.064***
	(0.015)	(0.011)	(0.011)	(0.008)
cutprcp(1,2]	−0.373***	0.277***	−0.177***	0.159***
	(0.026)	(0.023)	(0.022)	(0.016)
cutprcp(2,3]	−0.421***	0.399***	−0.215***	0.207***
	(0.037)	(0.042)	(0.032)	(0.031)
cutprcp(3,4]	−0.431***	0.395***	−0.220***	0.352***
	(0.050)	(0.047)	(0.042)	(0.042)
cutprcp(4, Inf]	−0.747***	0.715***	−0.383***	0.350***
	(0.066)	(0.073)	(0.062)	(0.050)
cuttrange(5,10]	−0.034	−0.076***	0.009	−0.026*
	(0.023)	(0.020)	(0.020)	(0.015)
cuttrange(10,15]	0.043	−0.165***	0.034	−0.072***
	(0.027)	(0.022)	(0.023)	(0.018)
cuttrange(15, Inf]	0.104***	−0.177***	0.063**	−0.072***
	(0.034)	(0.029)	(0.029)	(0.023)
cuthumid(-Inf,40]	−0.058*	0.038	−0.043*	0.031
	(0.031)	(0.026)	(0.026)	(0.020)
cuthumid(60,80]	−0.021	−0.005	−0.020	−0.015
	(0.019)	(0.015)	(0.014)	(0.010)
cuthumid(80, Inf]	−0.084***	0.039**	−0.050***	0.031**
	(0.024)	(0.019)	(0.018)	(0.013)
cutcloud(20,40]	−0.052***	0.021*	−0.034***	0.007
	(0.014)	(0.012)	(0.012)	(0.010)
cutcloud(40,60]	−0.086***	0.049***	−0.067***	0.036***
	(0.018)	(0.014)	(0.014)	(0.011)
cutcloud(60,80]	−0.142***	0.105***	−0.077***	0.057***
	(0.021)	(0.018)	(0.016)	(0.013)
cutcloud(80, Inf]	−0.200***	0.168***	−0.107***	0.077***
	(0.028)	(0.024)	(0.020)	(0.017)
City FE	Yes	Yes	Yes	Yes
Date FE	Yes	Yes	Yes	Yes
City:Year-Month FE	Yes	Yes	Yes	Yes
Observations	85,801	85,801	67,972	67,972
R^2	0.954	0.942	0.883	0.902
Adjusted R^2	0.951	0.939	0.877	0.897
Residual Std. Error	135.818	110.274	100.463	78.829

Note: *p<0.1; **p<0.05; ***p<0.01

Standard errors are in parentheses and are clustered on city-yearmonth and date.

Table D: User-level weather and expressed sentiment, Twitter all posts

	Dependent variable:	
	Positive	Negative
	(1)	(2)
cuttmax(-Inf,0]	−0.562***	0.080**
	(0.052)	(0.039)
cuttmax(0,5]	−0.465***	0.072*
	(0.043)	(0.037)
cuttmax(5,10]	−0.309***	0.058**
	(0.034)	(0.028)
cuttmax(10,15]	−0.189***	0.038*
	(0.025)	(0.020)
cuttmax(15,20]	−0.083***	0.006
	(0.020)	(0.014)
cuttmax(25,30]	−0.017	0.013
	(0.016)	(0.015)
cuttmax(30,35]	−0.096***	0.060***
	(0.023)	(0.019)
cuttmax(35, Inf]	−0.234***	0.212***
	(0.039)	(0.030)
cutprcp(0,1]	−0.073***	0.049***
	(0.012)	(0.009)
cutprcp(1,2]	−0.114***	0.114***
	(0.021)	(0.019)
cutprcp(2,3]	−0.155***	0.152***
	(0.034)	(0.030)
cutprcp(3,4]	−0.240***	0.252***
	(0.045)	(0.039)
cutprcp(4, Inf]	−0.330***	0.197***
	(0.060)	(0.041)
cuttrange(5,10]	0.025	−0.035**
	(0.019)	(0.016)
cuttrange(10,15]	0.057***	−0.066***
	(0.021)	(0.019)
cuttrange(15, Inf]	0.094***	−0.090***
	(0.028)	(0.024)
cuthumid(-Inf,40]	−0.027	0.030
	(0.027)	(0.024)
cuthumid(60,80]	0.006	−0.008
	(0.013)	(0.011)
cuthumid(80, Inf]	−0.016	0.028**
	(0.017)	(0.014)
cutcloud(20,40]	−0.052***	0.010
	(0.013)	(0.011)
cutcloud(40,60]	−0.072***	0.030**
	(0.014)	(0.012)
cutcloud(60,80]	−0.086***	0.035**
	(0.017)	(0.014)
cutcloud(80, Inf]	−0.116***	0.040**
	(0.021)	(0.017)
User FE	Yes	Yes
Date FE	Yes	Yes
City:Year-Month FE	Yes	Yes
Observations	81,388,085	81,388,085
R^2	0.129	0.110
Adjusted R^2	0.125	0.105
Residual Std. Error	32.702	25.976

Note: *p<0.1; **p<0.05; ***p<0.01
Standard errors are in parentheses and are clustered on city-yearmonth and date.

Table E: City-level weather and expressed sentiment, Twitter no weather terms

	Dependent variable:	
	Positive	Negative
	(1)	(2)
cuttmax(-Inf,0]	−0.621***	0.040
	(0.059)	(0.041)
cuttmax(0,5]	−0.435***	0.090***
	(0.040)	(0.033)
cuttmax(5,10]	−0.310***	0.099***
	(0.034)	(0.027)
cuttmax(10,15]	−0.201***	0.081***
	(0.024)	(0.019)
cuttmax(15,20]	−0.080***	0.037***
	(0.018)	(0.014)
cuttmax(25,30]	−0.010	0.010
	(0.018)	(0.014)
cuttmax(30,35]	−0.047*	0.034*
	(0.024)	(0.019)
cuttmax(35, Inf]	−0.178***	0.111***
	(0.036)	(0.028)
cutprcp(0,1]	−0.078***	0.054***
	(0.011)	(0.008)
cutprcp(1,2]	−0.154***	0.124***
	(0.022)	(0.015)
cutprcp(2,3]	−0.188***	0.139***
	(0.033)	(0.028)
cutprcp(3,4]	−0.194***	0.248***
	(0.045)	(0.040)
cutprcp(4, Inf]	−0.339***	0.162***
	(0.069)	(0.046)
cuttrange(5,10]	0.020	−0.028*
	(0.020)	(0.015)
cuttrange(10,15]	0.033	−0.076***
	(0.023)	(0.017)
cuttrange(15, Inf]	0.052*	−0.082***
	(0.029)	(0.022)
cuthumid(-Inf,40]	−0.031	0.024
	(0.025)	(0.019)
cuthumid(60,80]	−0.028**	−0.012
	(0.014)	(0.010)
cuthumid(80, Inf]	−0.055***	0.025*
	(0.018)	(0.013)
cutcloud(20,40]	−0.025**	0.006
	(0.012)	(0.010)
cutcloud(40,60]	−0.052***	0.032***
	(0.014)	(0.011)
cutcloud(60,80]	−0.051***	0.049***
	(0.016)	(0.013)
cutcloud(80, Inf]	−0.084***	0.070***
	(0.020)	(0.016)
City FE	Yes	Yes
Date FE	Yes	Yes
City:Year-Month FE	Yes	Yes
Observations	67,972	67,972
R^2	0.882	0.903
Adjusted R^2	0.876	0.898
Residual Std. Error	100.819	78.818

Note: *p<0.1; **p<0.05; ***p<0.01
Standard errors are in parentheses and are clustered on city-yearmonth and date.

Table F: User-level weather and expressed sentiment, Twitter no weather terms

	Dependent variable:	
	Positive	Negative
	(1)	(2)
cuttmax(-Inf,0]	−0.378***	−0.035
	(0.051)	(0.038)
cuttmax(0,5]	−0.293***	−0.002
	(0.041)	(0.037)
cuttmax(5,10]	−0.189***	0.014
	(0.032)	(0.028)
cuttmax(10,15]	−0.118***	0.010
	(0.024)	(0.020)
cuttmax(15,20]	−0.049***	−0.004
	(0.019)	(0.014)
cuttmax(25,30]	−0.010	0.005
	(0.016)	(0.014)
cuttmax(30,35]	−0.048**	0.025
	(0.023)	(0.019)
cuttmax(35, Inf]	−0.119***	0.121***
	(0.038)	(0.032)
cutprcp(0,1]	−0.043***	0.035***
	(0.012)	(0.009)
cutprcp(1,2]	−0.063***	0.063***
	(0.021)	(0.018)
cutprcp(2,3]	−0.089***	0.059**
	(0.033)	(0.028)
cutprcp(3,4]	−0.193***	0.132***
	(0.046)	(0.039)
cutprcp(4, Inf]	−0.235***	0.005
	(0.062)	(0.040)
cuttrange(5,10]	0.020	−0.034**
	(0.019)	(0.016)
cuttrange(10,15]	0.040*	−0.070***
	(0.021)	(0.019)
cuttrange(15, Inf]	0.063**	−0.102***
	(0.027)	(0.024)
cuthumid(-Inf,40]	−0.019	0.023
	(0.027)	(0.024)
cuthumid(60,80]	−0.002	−0.006
	(0.013)	(0.011)
cuthumid(80, Inf]	−0.013	0.020
	(0.017)	(0.014)
cutcloud(20,40]	−0.039***	0.008
	(0.013)	(0.011)
cutcloud(40,60]	−0.052***	0.028**
	(0.014)	(0.012)
cutcloud(60,80]	−0.054***	0.026*
	(0.016)	(0.014)
cutcloud(80, Inf]	−0.082***	0.037**
	(0.021)	(0.016)
User FE	Yes	Yes
Date FE	Yes	Yes
City:Year-Month FE	Yes	Yes
Observations	79,999,498	79,999,498
R^2	0.127	0.106
Adjusted R^2	0.123	0.102
Residual Std. Error	32.779	26.137

Note: *p<0.1; **p<0.05; ***p<0.01
Standard errors are in parentheses and are clustered on city-yearmonth and date.

Alternative measures of expressed sentiment

SentiStrength

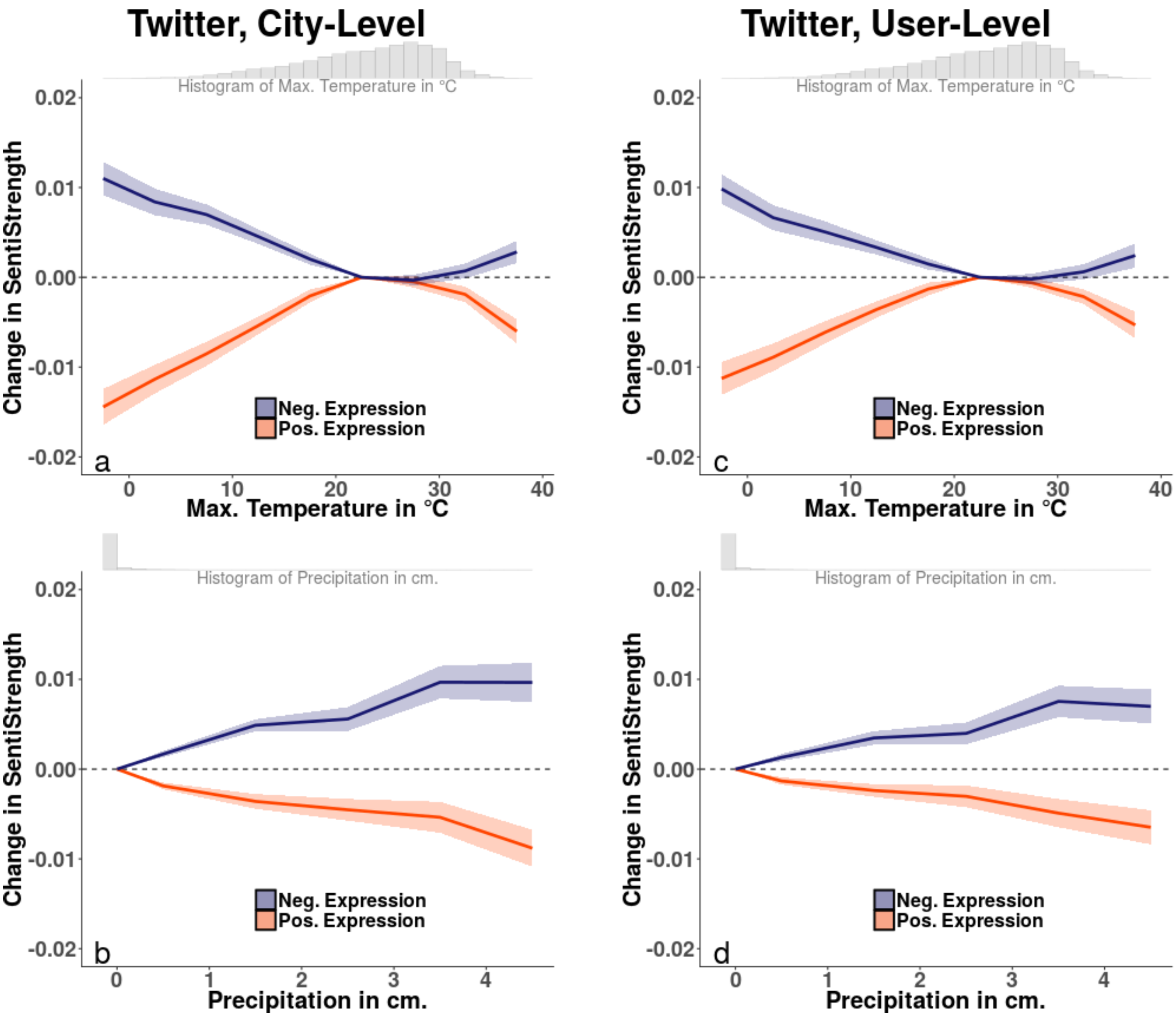

Fig G: **Replication using SentiStrength classification.**

In order to classify our Twitter data with the SentiStrength sentiment algorithm, we ran their local Java version (http://sentistrength.wlv.ac.uk/index.html). SentiStrength is designed to "estimate the strength of positive and negative sentiment in short texts, even for informal language". In Fig G and Fig H we replicate our results using the SentiStrength classifier.

Hedonometer

In order to classify our Twitter data with the Hedonometer sentiment algorithm, we employ their publicly available library (http://hedonometer.org/api.html), taking the city-day average across users' classified tweets. Hedonometer was built from a large corpus of words that were originally classified for sentiment by human workers. In Fig I and Fig J we replicate our results using the Hedonometer classifier. For this analysis, we code Hedonometer scores below 4 as negative and scores above 6 as positive.

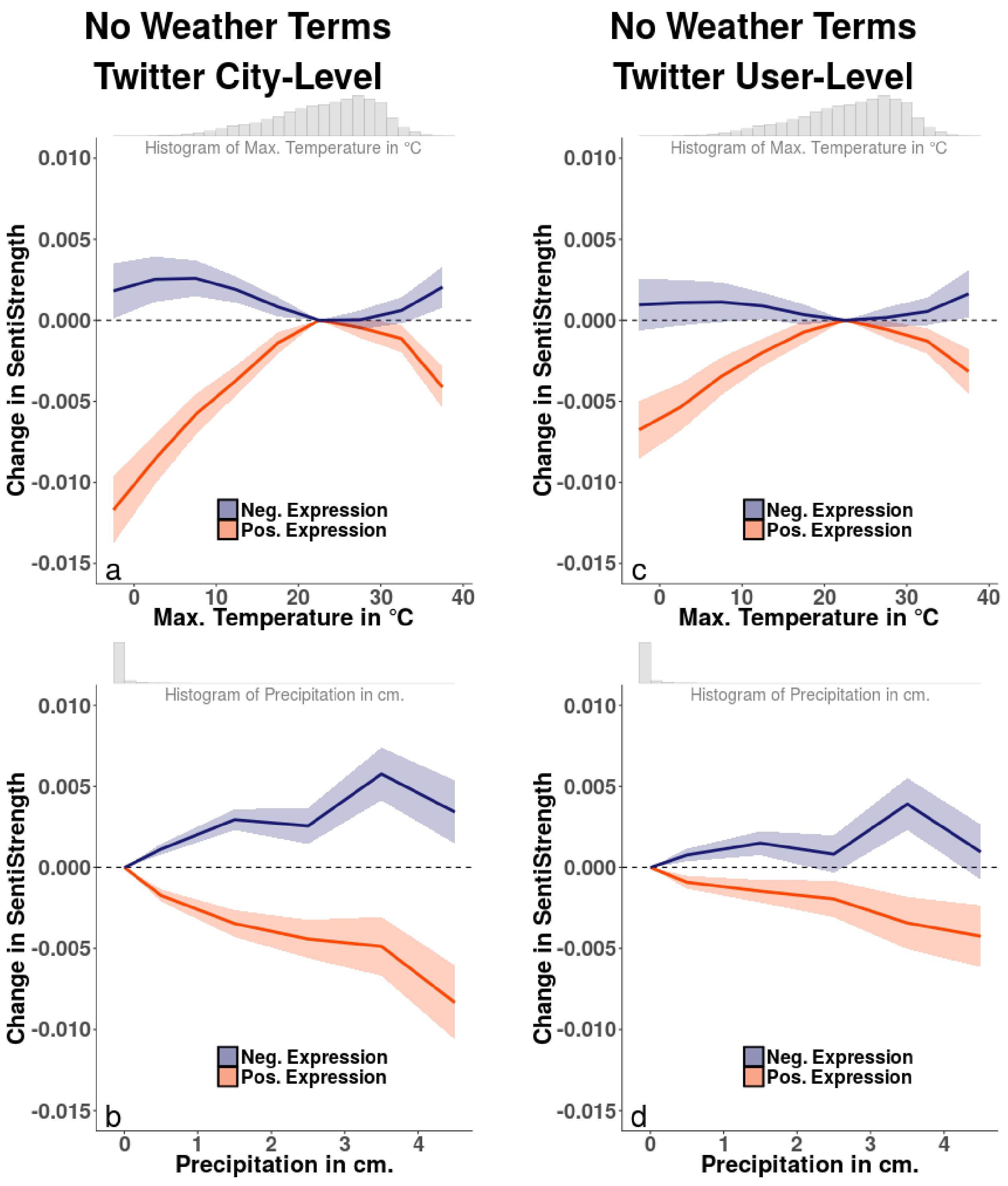

Fig H: **Replication using SentiStrength classification.**

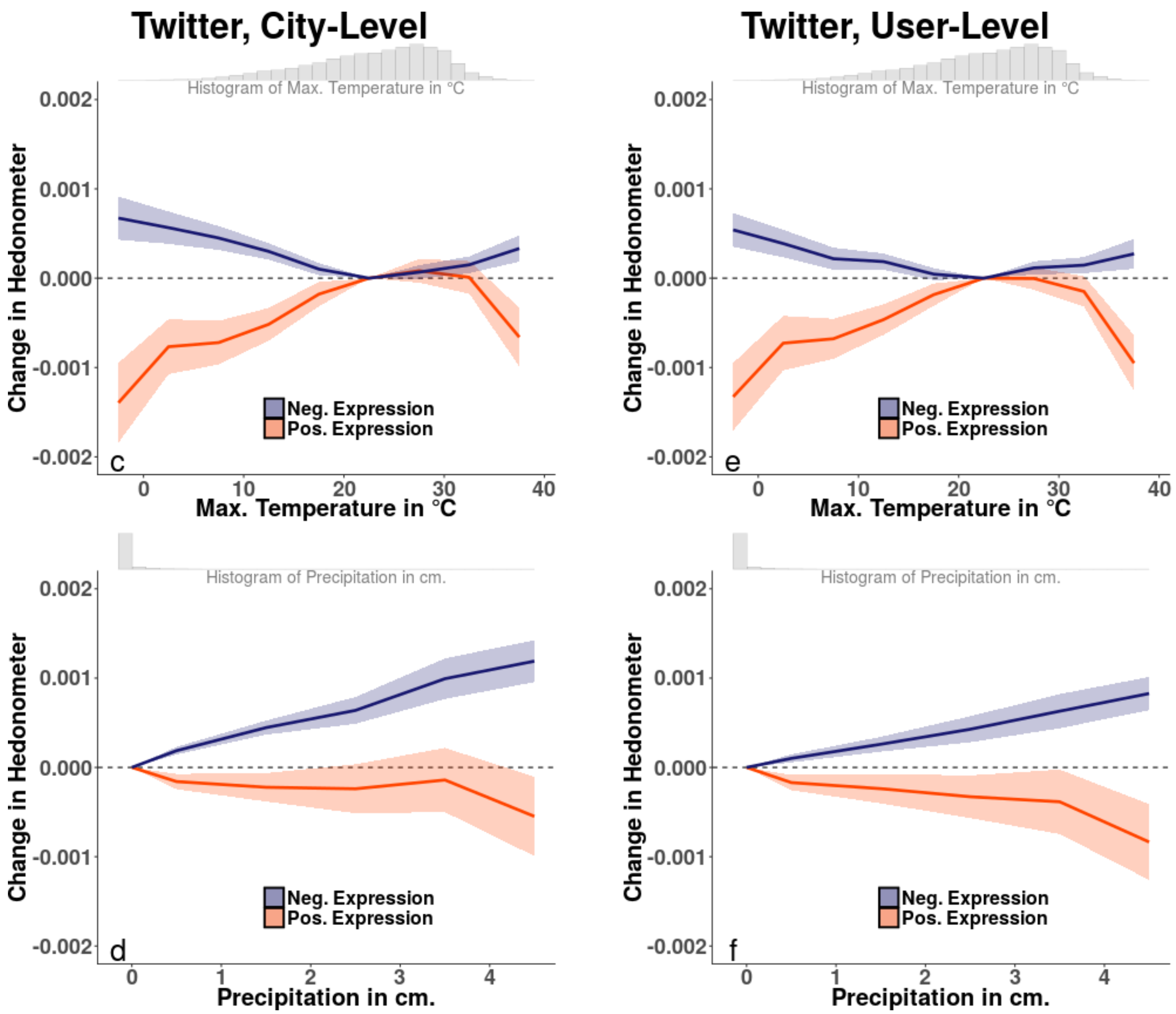

Fig I: **Replication using Hedonometer classification.**

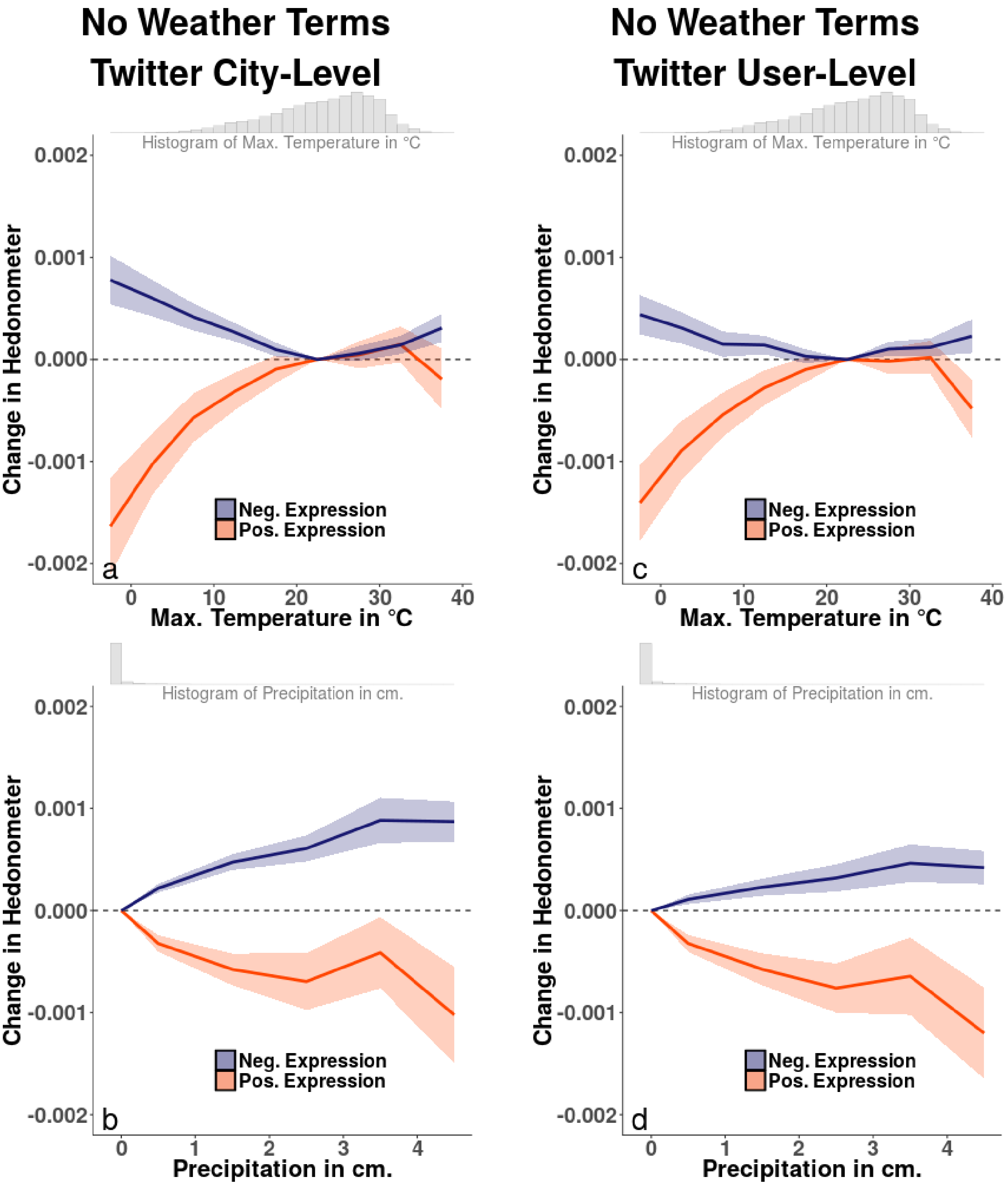

Fig J: **Replication using Hedonometer classification.**

Correlation of classifiers of expressed sentiment

Fig K displays the positive correlation observed between our positive metrics of expressed sentiment: LIWC, SentiStrength, and Hedonometer. The Hedonometer metric is moderately positively correlated with the other two, but exhibits notably less correlation than LIWC and SentiStrength exhibit between one another.

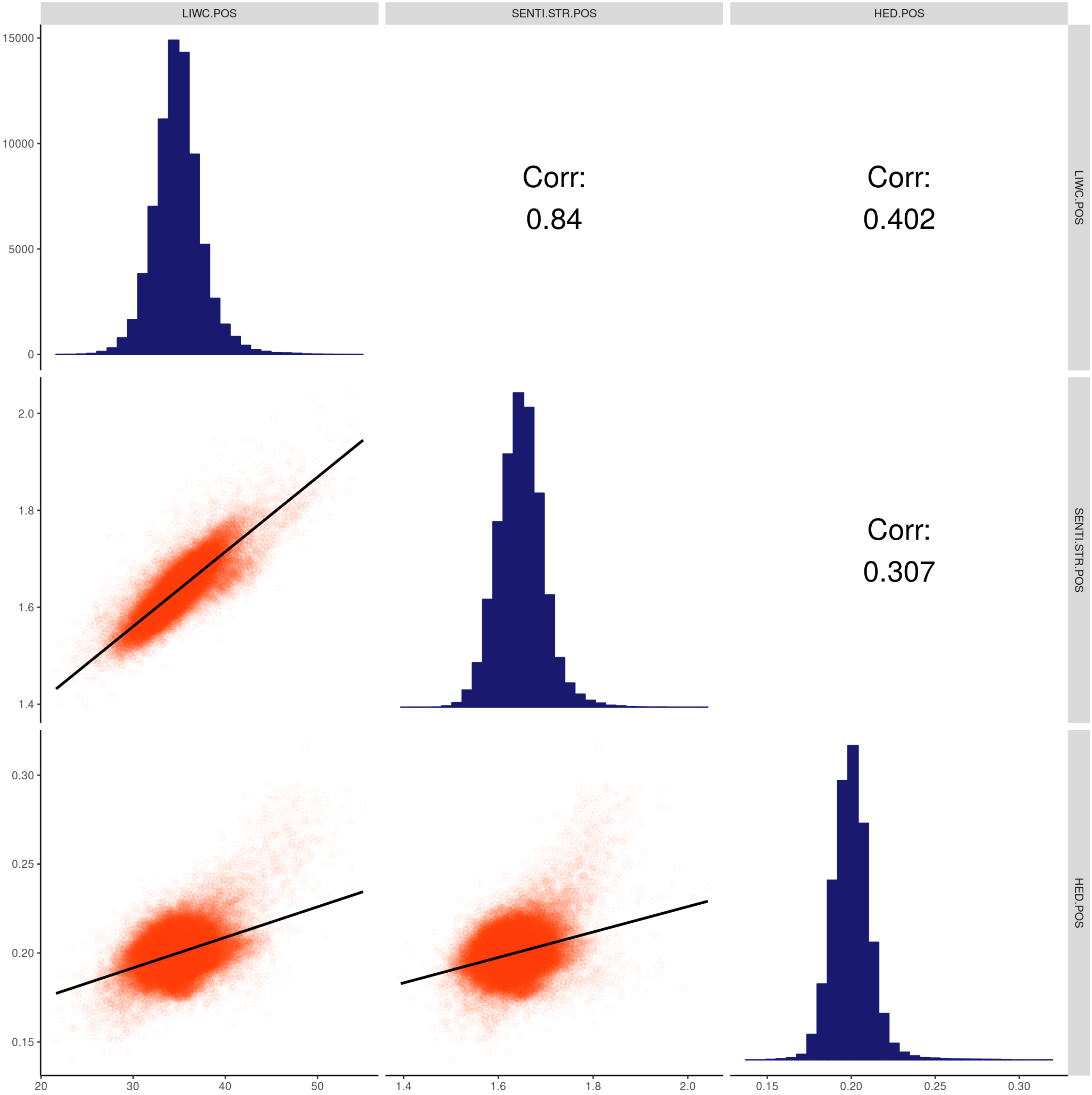

Fig K: **Correlation between alternative positive sentiment state metrics.**

Fig L displays the positive correlation observed between our negative sentiment classification metrics As can be seen, all three metrics share high correlations on their classification of negative expressed sentiment.

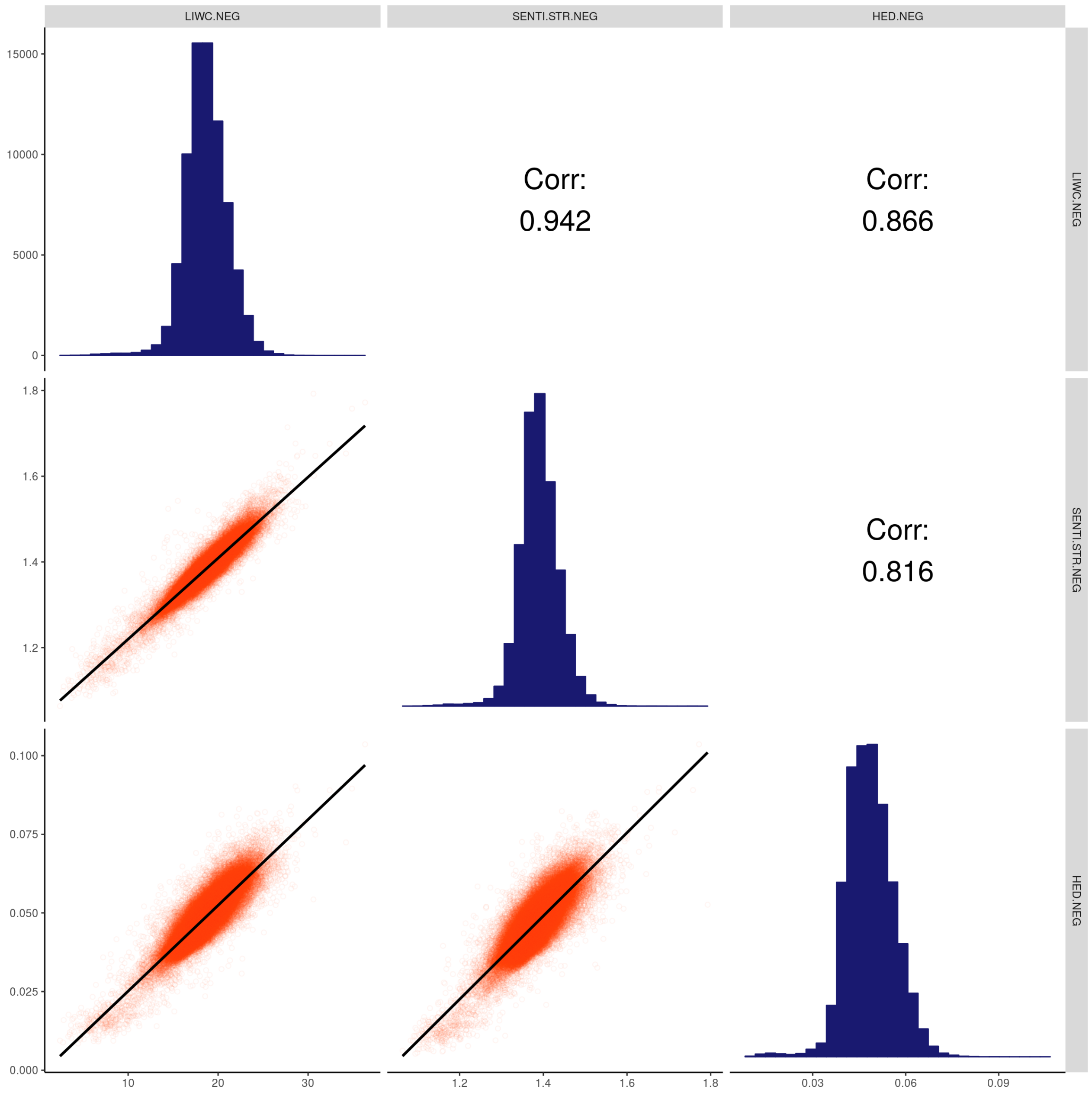

Fig L: **Correlation between alternative negative expressed sentiment metrics.**

References

1. Wooldridge JM. Econometric analysis of cross section and panel data. MIT press; 2010.
